# Preliminary Data on Post Market Safety Profiles of COVID 19 Vaccines in Rheumatic Diseases: Assessments on Various Vaccines in Use, Different Rheumatic Disease Subtypes, and Immunosuppressive Therapies: A Two-Centers Study

**DOI:** 10.3390/vaccines9070730

**Published:** 2021-07-02

**Authors:** Cinzia Rotondo, Francesco Paolo Cantatore, Marco Fornaro, Ripalta Colia, Giuseppe Busto, Valeria Rella, Stefania Sciacca, Lucia Lops, Daniela Cici, Nicola Maruotti, Francesca D’Onofrio, Florenzo Iannone, Addolorata Corrado

**Affiliations:** 1 Rheumatology Unit, Department of Medical and Surgical Sciences, Ospedali Riuniti di Foggia, Viale Luigi Pinto 1, 71122 Foggia, Italy; francescopaolo.cantatore@unifg.it (F.P.C.); ripaltacolia@gmail.com (R.C.); giuseppe.busto@unifg.it (G.B.); valeria.rella92@gmail.com (V.R.); stefania.sciacca@hotmail.it (S.S.); lucialops85@gmail.com (L.L.); daniela.cici@gmail.com (D.C.); nicola.maruotti@unifg.it (N.M.); drfrancesca.donofrio@gmail.com (F.D.); ada.corrado@unifg.it (A.C.); 2 Rheumatology Unit, Department of Emergency and Organ Transplantations, Policlinico, Piazza G. Cesare 11, 70124 Bari, Italy; marco3987@hotmail.it (M.F.); florenzo.iannone@uniba.it (F.I.)

**Keywords:** rheumatic diseases, relapse, flare, disease activity, DMARDs, biologic drugs, safety, efficacy, anti-COVID-19 vaccine, comirnaty, vaxzevria, SARs-COV-2

## Abstract

An increased risk of developing severe infections has been evidenced in rheumatic disease (RD) patients, and anti-COVID-19 vaccination is strictly recommended for RD patients. However, up to now, no data are available on safety, immunogenicity and efficacy of COVID-19 vaccinations in RD patients. The possible development of adverse events (AEs), including the flare-up of underlying RD, represents a matter of growing importance. The aim of our study is to assess, in RD patients, the safety profile of different types of approved vaccines and the possible influence of immunosuppressive therapies and clinical or demographic characteristics of RD patients on development of AEs. Participants (*n* = 185; 30.7%) received anti-COVID-19 vaccinations, 137 with autoimmune/chronic inflammatory RD (Au/cIn-RD) and 48 with nonautoimmune/chronic inflammatory RD (no-Au/cIn-RD). AEs were recorded in 42% of patients after the first dose of vaccine, and in 26% of patients after the second dose. The most common reported AEs after anti-COVID 19 vaccines were site injection pain (17%), headache (12%), fever (12%), myalgia (10%) and fatigue (10%). Relapses of the underlying Au/c-In-RD were recorded in 2.2% of patients after the first dose of vaccine. In Au/c-In-RD the risk of developing AEs after the first dose of vaccine was lower in older patients (OR = 0.95; *p* = 0.001), and in the group of patients with complete control of RD (OR: 0.2; *p* = 0.010). A lower percentage of AEs was observed in patients with complete control of their Au/cIn-RD (29%) compared to those with low (57%) or moderate-high disease activity (63%) (*p* = 0.002 and *p* = 0.006 respectively). In this study all types of COVID-19 vaccines in use in Italy seemed safe in RD patients. The results of this study might provide reassuring information for Au/cIn RD patients and clinicians and could strengthen the data on vaccine safety to guide the use of COVID-19 vaccines in Au/cIn-RD on immunosuppressive agents.

## 1. Introduction

Due to the lack of appropriate and effective treatments for COVID-19, vaccination seems to be only reliable preventive method to avoid hospitalization and severe complications of SARS-COV 2 infection [1]. Even though there is enormous competition regarding the development, production and commercialization of several vaccines, few vaccines are currently approved, and some of these vaccines are in use in limited part of the world. Actually, three types of anti-COVID-19 vaccines are approved by the AIFA (Agenzia Italiana del Farmaco) for the usage in Italy: Comirnaty (BioNTech/Pfizer—Germany/USA), Moderna (USA), and Vaxzevria (AstraZeneca—England/Sweden), although Comirnaty and Vaxzevria are more widely used in Italy [2].

Autoimmune/chronic-inflammatory rheumatic diseases (Au/cIn-RD), such as connective tissue diseases, vasculitis, rheumatoid arthritis, psoriatic arthritis and spondylarthritis affect at least 1–2% of the global population [3,4]. An increased risk of developing severe infections caused by immune system dysregulation and use of immunosuppressive drugs has been evidenced in Au/cIn-RD patients [5,6,7,8,9,10,11,12]. Therefore, practicing preventive measures is strongly suggested during COVID-19 pandemic in global population as well as in Au/In- RD patients [13,14,15]. In addition, COVID-19 vaccination is strictly recommended for these patients, as for common people, from many rheumatology associations. The American College of Rheumatology (ACR) has recently proposed recommendations for COVID-19 vaccination in RD patients and a practical guidance for immunosuppressive treatment management during the period of immunization. However, up to now, no data are available on safety, immunogenicity and efficacy of COVID-19 vaccinations in autoimmune RD patients on immunomodulatory treatments, due to the exclusion of these patients from clinical trials of COVID-19 vaccines. So, safety of new mRNA vaccines is a main concern in patients with autoimmune diseases, especially for the potential effect of vaccine to trigger a flare-up of underlying Au/cIn-RD. Nonlive vaccines seem to be safer in Au/cIn RD patients, but no data are available on the safety of vaccines based on mRNA in these patients, due to the fact that it is the first time the mRNA technology is being used for vaccine production [16].

In this new context, we present a study on the safety of anti-COVID-19 vaccines in RD patients approved for use in Italy, assessing the impact of vaccine type, immunosuppressive treatment, RD subsets and the clinical and demographic characteristics of patients.

## 2. Materials and Methods

A questionnaire inquiring whether RD patients had undergone COVID-19 vaccination, and if adverse events (AEs) had developed, was administrated to 325 outpatients with different RDs at the Rheumatology Units of two University Hospitals in Apulia, with sites in Foggia and in Bari. We also evaluated the compliance of treatment management during immunization with COVID-19 vaccine as in the clinical guidance summary for RD patients proposed by the ACR [16].

### 2.1. Patients

All RDs were included in the study. We divided the patients enrolled into two groups: those with Au/cIn-RD, such as juvenile idiopathic arthritis (JIA), rheumatoid arthritis (RA), psoriatic arthritis (PsA), undifferentiated spondylarthritis (SpA), undifferentiated connective tissue disease (UCTD), systemic erythematous lupus (SLE), systemic sclerosis, Sjogren’s syndrome (SS), mixed connective tissue disease (MCTD), inflammatory myositis (IM)), and vasculitis; and those with nonautoimmune/noninflammatory RD (no-Au/cIn-RD), such as fibromyalgia, osteoporosis, and osteoarthritis. The presence of comorbidities was summarized with the Charlson corbidity index (CCI) [17].

The inclusion criteria were subjects more than 18 years old, at more than 24 days after vaccination, and with the presence of RD.

### 2.2. Vaccination

All patients were immunized according to the schedule time of each vaccine in use. In line with World Health Organization (WHO), AEs following immunization were classified as minor reactions (occurring within a short time of administration, complete resolution in a short period, can be local (pain, swelling or redness at administration site) or systemic (fever, malaise, muscle pain, headache, or appetite loss), and severe reactions (can be disabling and rarely life-threatening, including serious reactions such as death, inpatients hospitalization, persistent or significant disability or life-threatening) [18].

Demographic characteristics, clinical data, RD subsets, vaccine types, development of AEs (including disease relapse) and the presence of comorbidities were collected. The disease activity at the vaccine administration time was synthetized as remission, low disease activity and moderate to high disease activity. Serological tests for detection of antispike protein antibodies were performed at referral laboratories of each patient after at least four weeks from the last vaccine dose administration, and the cut-off values recommended by commercially available assays.

This study followed ethical standards and the principles of Helsinki declarations. The local ethics committee approved this study with protocol number CE 49.19. All patients gave written informed consent about the nature and aim of the study, including consent to publish data.

### 2.3. Statistical Analysis

The results are expressed as mean ± standard deviation, and categorical variables are expressed as absolute number and percentage in each category. The data normality was assessed using the Shapiro–Wilk’s test. We compared the clinical and laboratory data of study groups of patients using Student’s *t* test. The comparisons among multiple groups were assessed by analysis of variance (ANOVA), followed by post hoc analysis with Bonferroni multiple testing correction where appropriate. The comparisons between categorical variables were performed by the Pearson chi-square or Fisher’s exact test, as appropriate. The evaluation of risk to develop AEs, by means of odds ratio, was assessed using binary logistic regression analysis and was expressed as the odds ratio (OR) and 95% confidence interval (CI). *p* ≤ 0.05 was defined as statistically significant. Statistical analysis was performed using IBM SPSS Statistics 23 (Armonk, NY, USA: IBM Corp.).

## 3. Results

Among the 601 interviewed subjects, 185 (30.7%) participants received anti-COVID-19 vaccination (140 females and 45 males). Mean age of vaccinated participants was 60.2 ± 14.2 years (range 20–85 years), and RD mean duration was 7.6 ± 7.3 years (range 1–37 years). Of the participants, 41 (22%) received the AstraZeneca COVID-19 vaccine and 144 (78%) the BioNTech-Pfizer COVID-19 vaccine. Just seven (17%) patients immunized with the AstraZeneca vaccine and 83 (57%) patients with BioNTech-Pfizer vaccine received two doses. A comparison between the demographic and clinical characteristics of groups of patients in the study are described in Table 1. Patients with Au/cIn-RD compared to those with no-Au/cIn-RD were younger (57.0 years ± 14.0 vs. 66.8 years ± 12.8; *p* = 0.0001), received the second dose of vaccine with higher rate (43.5% vs. 9.5%; *p* = 0.005), and had a lower Charlson corbidity index score (CCI) (0.5 ± 1 vs. 1.4 ± 1.6; *p* = 0.009) (Table 1).

In addition, the description of subgroups of patients stratified for types of vaccines received and for RD are shown in Table 2. Less patients immunized with AstraZenca vaccine received two doses of vaccines than those who received the BioNTech-Pfizer vaccine (0.0001) (Table 2).

AEs were recorded in 77 (42%) patients after the first dose of vaccine, and in 24 (26%) patients after the second dose. In particular, seven patients developed AEs just after the second dose of vaccine, and 13 patients after both doses of vaccine.

No severe reactions, according to WHO (World Health Organization) classification, were described after either dose. All patients reported that AEs disappeared in 48 h. In all group of patients in the study, the most common reported AEs after the first dose of anti-COVID 19 vaccines were site injection pain (17%), headache (12%), fever (12%), myalgia (10%), and fatigue (10%). A similar trend occurred after the second dose of vaccine, fever (15%), myalgia (10%), site injection pain (9%), headache (4%) and fatigue (2%).

The occurrence of relapsing of underlying RDs were recorded in 2.2% of patients after the first dose of anti-COVID-19 vaccine; all these patients received the BioNTech-Pfizer vaccine, although no significant differences in AEs between the BioNTech-Pfizer and AstraZeneca vaccines were found (Table 2). No relapse of RD was observed after the second dose of vaccine.

RD patients with previous SARs-COV-2 infections did not present significant differences in occurrence of AEs after the first or the second dose compared to noninfected RD patients. All these patients were vaccinated after at least 90 days from the negative SARs-COV-2 nasal swab.

By univariate regression analysis, in RD the risk to develop AEs after the first dose of vaccine was lower in older patients (OR = 0.95; 95%CI 0.92–0.97; *p* = 0.001), and in the group of patients with complete control of RD (OR: 0.2, 95%CI: 0.08–0.76; *p* = 0.010). The risk of developing AEs after the second dose was lower in older patients (OR: 0.95; 95%CI 0.92–0.99; *p* = 0.012), and in a group of patients in RD remission (OR: 0.125, 95%CI 0.025–0.630; *p* = 0.012) and was higher in patients who developed AEs after the first dose (OR: 5.1, 95%CI: 1.8–14; *p* = 0.002).

### 3.1. Rheumatic Disease Activity and Adverse Events after Anti-COVID 19 Vaccinations

**Figure 1:**
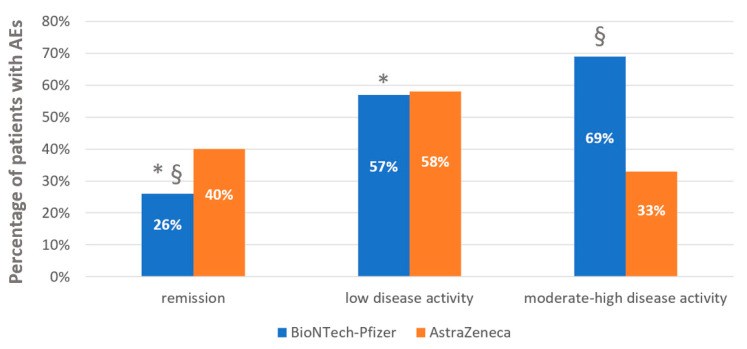
Percentages of adverse events after the first dose of vaccine in autoimmune/chronic inflammatory rheumatic diseases (Au/cIn-RD) stratified for different vaccines in use and disease activity. Data are shown as %. Statistical significance was set at *p* ≤ 0.05. AEs: adverse events. Number of patients immunized with BioNTech-Pfizer: remission 53, low disease activity 38, moderate-high disease activity 16. Number of patients immunized with AstraZeneca: remission 15, low disease activity 12, moderate-high disease activity 3. AEs in remission patients immunized with BioNTech—Pfizer vs. AstraZeneca *p* = 0.239. AEs in low disease activity patients immunized with BioNTech–Pfizer vs. AstraZeneca *p* = 0.597. AEs in moderate-high disease activity patients immunized with BioNTech–Pfizer vs. AstraZeneca *p* = 0.296. AEs in patients immunized with BioNTech–Pfizer: * remission vs. low disease activity *p* = 0.001; § remission vs. high disease activity *p* = 0.001; low disease activity vs. moderate-high disease activity *p* = 0.567. Patients immunized with AstraZeneca: remission vs. low disease activity *p* = 0.566, remission vs. moderate-high disease activity *p* = 0.564, low disease activity vs. moderate-high disease activity *p* = 0.745.

Considering the disease activity of Au/cIn-RD, 68 patients were in complete remission, 49 had low disease activity, and 19 patients had moderate-high disease activity. The percentage of recorded AEs after the first dose of vaccine was significantly higher in patients with low disease activity (57%) and in those with moderate to high disease activity (63%) compared to patients in remission (29%) (*p* = 0.002 and *p* = 0.006 respectively). Among patients receiving the second dose of vaccine, the AEs were recorded in rates significantly higher in patients with low disease activity (45%) and in those with moderate to high disease activity (50%) compared to patients in remission (11%) (*p* = 0.002 and *p* = 0.001 respectively). All patients that developed AEs after the second dose of vaccine were immunized with the BioNTech-Pfizer vaccine, although no significant differences were found between BioNTech-Pfizer and AstraZeneca (Table 2). The percentages of AEs after the first dose stratified for disease activity and vaccines are shown in Figure 1.

### 3.2. Adverse Events after Anti-COVID-19 Vaccinations in Autoimmune/Inflammatory Rheumatic Diseases and in Nonautoimmune/Noninflammatory Rheumatic Diseases

Among 185 vaccinated subjects, 107 had arthritis (1% JIA, 26% RA, 50% PsA, 23% SpA), 24 had connective tissue diseases (13% IM, 8% SLE, 25% SS, 21% SSc, 33% UCTD), 6 vasculitis, 20 osteoporosis, 9 fibromyalgia, and 19 osteoarthrosis (Table 1).

No significant differences in occurrence of AEs after the first and the second dose of vaccine between groups of patients with Au/In-RD (respectively 44% and 28%) and those with No-Au/In-RD (respectively 36% and 19%) were observed (respectively, *p* = 0.341 and *p* = 0.403).

Regarding the different subsets of Au/cIn-RD, 48 (62%) patients with arthritis, 9 (12%) patients with connective tissue diseases, and 3 patients with vasculitis developed AEs after the first dose of vaccine. Fifteen patients (62%) with arthritis, and five (21%) with connective tissue diseases developed AEs after the second dose of vaccine.

Considering Au/In-RD patients, no significant differences were found in occurrence of AEs between groups immunized with AstraZeneca or BioNTech-Pfizer after the first dose (respectively 46% vs. 43%, *p* = 0.750) and after the second dose (respectively 0% vs. 31%; *p* = 0.109).

By binary regression analysis, the risk of AEs seems to be independent of the presence of Au/cIn-RD.

### 3.3. Adverse Events after Anti-COVID-19 Vaccinations and Immunosuppressive Drugs

Sixty (44%) Au/cIn-RD patients were treated with cs-DMARDs, 57 (42%) with b-DMARDs or ts-DMARDs, one with IVIG, 37 (27%) with steroids (mean dose 3.9 ± 1.2 mg prednisone equivalent). In particular, among the mentioned patients 34 (30%) received a combination treatment with two or more immunosuppressive drugs among steroids, cs-DMARDs and b/ts-DMARDs (Table 1).

Considering patients with Au/cIn-RD treated with immunomodulatory drugs, AEs after the first dose of vaccine were recorded in 25 (44%) treated with b/ts-DMARDs, in 26 (43%) treated with cs-DMARDs, and in 15 (40%) treated with CS. After the second dose, AEs were recorded in seven (22%) patients treated with b/ts-DMARDs, four (25%) treated with CS, and nine (27%) treated with cs-DMARDs.

No differences were observed in the rate of AEs between the group of Au/cIn-RD patients treated with combo therapy (44%) and the group of patients treated with mono-therapy (41%), (*p* = 0.777) after the first dose of vaccine. Similar findings were observed after the second dose, respectively 21% vs. 25% (*p* = 0.739).

In addition, no significative differences were found in AEs occurring in different types of vaccines, BioNTech-Pfizer and AstraZeneca, after the first (*p* = 0.079) and after the second dose (*p* = 0.072) of the vaccines (data not shown).

By binary regression analysis, the risk of AEs seemed to be independent of the use of immunosuppressive drugs (cs-DMARDs, b/ts-DMARDs or steroids).

### 3.4. Immunosuppressive Drugs Management before and after Anti-COVID-19 Vaccination, Adherence to International Treatment Recommendations and Antibodies Response

Regarding immunosuppressive treatment management before and after immunization, just 70 (51%) Au/cIn-RD patients complied with the COVID-19 vaccine clinical guidance of the ACR for patients with rheumatic and musculoskeletal diseases [16].

Ten (5%) of patients with Au/cIn-RD dosed serum levels of antispike antibodies performed at their local-referral laboratories. The mean serum level of antispike antibodies was 1638.3 ± 857.1 U/mL. In all these patients the serum levels of antispike antibodies were higher than the normal range.

No significant differences in serum levels of antispike antibodies between patients in which immunosuppressive drugs were disrupted, and in those who continued treatment, were found (respectively, 1800 ± 955.6 U/mL vs. 1261 ± 511.2 U/mL; *p* = 0.394).

## 4. Discussion

Patients with Au/cIn RD have a higher risk of infection and are prone to develop more severe forms of infections due to underlying autoimmune dysregulation, and immunosuppression induced by treatments such as steroids, cs-DMARDs, b-DMARDs and ts-DMARDs [5,6,7,8,9,10,11,12]. Since the vaccinations prevent severe infectious diseases by means of induction and strengthening the immune response, vaccinations are particularly recommended in Au/cIn RD patients, as suggested by the EULAR (European League Against Rheumatism) in 2019 [10]. Immunocompromised patients seem to exhibit increasing susceptibility to develop severe forms of COVID-19 with higher mortality rates. Nonlive vaccines seem to be safer in Au/cIn RD patients, but no data are available on safety of vaccines based on mRNA in these patients, due to the fact that it is the first time mRNA technology is being used for vaccine production. Therefore, rapid development and approval time of anti-COVID-19 vaccines has precluded the assessment of efficacy and safety in particular subgroups of the population, such as patients with autoimmune diseases. In this way, the recommendations on COVID-19 vaccination in Au/cIn RD are structured only on hypothetical bases [16].

In our study, the safety of all vaccines approved in Italy was generally comparable between patients with Au/cIn RD treated with either b/ts-DMARDs or cs-DMARDs, and those with no-Au/cIn-RD. Similar results on safety were recorded irrespective of previous COVID-19 infected patients and those not infected.

The first and the second doses of all types of vaccines considered in this study were well tolerated in Au/cIn-RD patients and in non-Au/cIn-RD. The majority of AEs recorded were site injection pain (17%), headache (12%), fever (12%), myalgia (10%) and fatigue (10%), in accordance with a previously published study on the general population [19,20]. We underline that thrombosis events were not observed in our study population.

Regarding disease activity of Au/cIn RD and vaccinations, in 2019 the EULAR recommendation suggested promotion of vaccinations in quiescent disease phases [21] to avoid disease flare-up and to favor a good immune response. However, due to SARS-CoV-2’s severity, a rapid anti-COVID-19 immunization was strongly suggested, and the patients included in this study were vaccinated in different activity phases of their disease. We found that patients in complete control of their RD had a lower risk (OR: 0.2, 95%CI: 0.08–0.76; *p* = 0.010) in developing AEs. A lower AEs percentage was found in groups of patients in remission compared to those with low or moderate to high disease activity, validating, also for anti-COVID-19 vaccination, a previous EULAR recommendations to vaccinate preferably in a quiescent disease phase [21].

Concerning the controversial debate on the possibility of inducing or enhancing the autoimmune response by means of molecular mimicry between the viral antigen and host antigen [22,23], as previously evidenced in hepatitis B vaccinations [24], in our study we evidenced a low rate (5.7%) of disease relapse of Au/cIn-RD after the first dose of vaccine, as previously reported for influenza vaccines [25,26,27]. The rate of RD flare-up observed after BioNTech-Pfizer vaccination could be due to the highest frequency of this vaccine administration, although no significant differences in AEs between BioNTech-Pfizer and AstraZeneca were found (Table 2).

A combination of immunosuppressive drugs seems not interfere with the occurrence of AEs in Au/cIN-RD. These data were not described in previous published studies.

The higher risk of developing AEs after the second dose of the anti-COVID-19 vaccine in the group of patients that experienced AEs after the first dose of vaccine, could be due to particular individual susceptibilities, regardless of RD, immunosuppressive agents and disease activity phases.

The Italian health system suggests immunization of previously infected patients after at least three months of COVID-19 healing. In our study, patients previously infected with COVID-19 were vaccinated 90 days after a negative nasal swab. We underline that no AEs were recorded in RD patients with previous COVID-19.

Regarding efficacy of immunization, there are few available data; however, we noted a good immune response after vaccination in Au/cIn-RD patients who dosed antispike antibodies.

This study has some limitations. First, heterogeneous patients with RD on different immunosuppressive treatments were enrolled, so no particular information on specific autoimmune diseases and b/ts-DMARDs treatment is provided. Second, after COVID-19 vaccination, antibody detection was lacking in a large proportion of patients. The immune response and the persistence of antibody titer after immunization in patients with Au/cIn RD treated with different immunosuppressive drugs should be clarified to define optimal treatment management in these patients. Further studies on the efficacy of different types of COVID vaccines could provide additional information. Another matter that could interfere with the results of this work is the small sample size of our cohorts, which might reduce the statistical power of several analyses.

## 5. Conclusions

Due to the severity of SARs-COV-2, anti-COVID-19 vaccination is strongly recommended in the global population, as well as in people with Au/cIn-RD. In this study all types of COVID-19 vaccines in use in Italy seemed safe in RD patients. The results of this study might provide reassuring information for Au/cIn RD patients and clinicians and could strengthen the data on vaccine safety to guide the use of COVID-19 vaccines in Au/cIn-RD patients on immunosuppressive agents.

## Figures and Tables

**Table 1:** Comparison of demographic and clinical characteristics between patients with autoimmune/chronic inflammatory rheumatic diseases (Au/cIn-RD) and those with nonautoimmune/non chronic inflammatory rheumatic diseases (no-Au/cIn-RD).

Characteristics		Au/cIn-RD *n* = 137	Non-Au/cIn-RD *n* = 48	*p*-Value
Age (years)		57.0 ± 14.0	66.8 ± 12.8	0.0001
Sex female/male *n* (%)		97 (70%)/40 (30%)	43 (89%)/5 (11%)	0.202
Disease duration duration (years)		9 ± 7.6	3.6 ± 5.9	0.207
BMI		26.3 ± 5.4	26 ± 4.3	0.765
Arthritis *n* (%)		107 (78%)		
Connective tissue diseases *n* (%)		24 (18%)		
Vasculitis *n* (%)		6 (4%)		
Osteoporosis *n* (%)			20 (41%)	
Osteoarthrosis *n* (%)			19 (40%)	
Fibromyalgia *n* (%)			9 (19%)	
Steroid use *n* (%)		37 (27%)		
Prednisone equivalent (mg)		3.9 ± 1.2		
cs-DMARDs *n* (%)		60 (43%)		
cs-DMARDs *n* (%)	MTX	42 (70%)		
	SSZ	1 (2%)		
	MMF	6 (10%)		
	HCQ	9 (15%)		
	colchicine	2 (3%)		
b/ts-DMARDs *n* (%)		57 (42%)		
b/ts-DMARDs *n* (%)	Anti-TNF alpha	29 (51%)		
	Anti-IL17	11 (20%)		
	Anti-IL6	6 (10%)		
	Jak-i	6 (10%)		
	Abatacept	5 (9%)		
IgIV *n* (%)		1 (%)		
Combo therapy		34 (30%)		
Vaccines	AstraZeneca	30 (22%)	11 (23%)	0.986
	BioNTech/Pfizer	107 (78%)	37 (77%)	0.990
Vaccine doses	One dose	66 (48%)	29 (60%)	0.157
	Second dose	71 (52%)	19 (40%)	0.198
Disease activity	Remission	68 (50)%		
	Low	50 (36%)		
	Moderate-high	19 (14%)		
CCI		0.67 ± 1	1.8 ± 1.6	0.036

Data are shown as mean ± sd or *n* (%). Statistical significance was set at *p* ≤ 0.05. AEs: adverse events; BMI: body mass index; b/ts-DMARDs: biologic/target synthetic-Disease Modifying Antirheumatic Drugs; CCI: Charlson Comorbidity index; COVID-19: Corona Virus Disease; cs-DMARDs: conventional synthetic- Disease Modifying Antirheumatic Drugs; IgIV: Intravenous immunoglobulin; IL: interleukin; JAK-i: Janus kinase inhibitors; MMF: mofetil mycophenolate; MTX: methotrexate; SSZ: sulfasalazine; TNF: tumor necrosis factor.

**Table 2:** Comparison of demographic and clinical characteristics among different vaccines in use in Italy stratified for autoimmune/chronic inflammatory rheumatic diseases (Au/cIn-RD) and nonautoimmune/non chronic Inflammatory rheumatic diseases (no-Au/cIn-RD). Data are shown as mean ± sd or *n* (%). Statistical significance was set at *p* ≤ 0.05. AEs: adverse events; CCI: Charlson Comorbidity index; COVID-19: Corona Virus Disease; cs-DMARDs: Conventional Synthetic Disease-Modifying Antirheumatic Drugs; b/ts-DMARDs: biologic/target synthetic Disease-Modifying Antirheumatic Drugs.

Title	AstraZeneca	BioNTech-Pfizer	*p*-Value
**Au/cIn-RD**	***n* = 30**	***n* = 107**	
Age (years)	59.2 ± 12.4	57.4 ± 14.2	0.532
Sex female/male *n*	21/9	76/31	0.950
Disease duration (years)	6.9 ± 5.9	9.5 ± 7.5	0.090
BMI	25 ± 4.6	26.8 ± 5.8	0.118
Steroid use *n* (%)	9 (30%)	28 (26%)	0.674
Prednisone equivalent (mg)	3.3 ± 1.2	4.5 ± 1.8	0.775
cs-DMARDs *n* (%)	13 (53%)	47 (44%)	0.108
b/ts-DMARDs *n* (%)	8 (27%)	49 (46%)	0.145
Combo immunosuppressive therapy	8 (27%)	26 (29%)	0.858
Vaccine second dose	6 (20%)	65 (60%)	0.001
Arthritis *n* (%)	25 (83%)	82 (76%)	0.826
Connective tissue diseases *n* (%)	5 (17%)	19 (18%)	0.421
Vasculitis *n* (%)	0 (0%)	3 (3%)	-
Polymyalgia Rheumatica *n* (%)	0 (0%)	3 (3%)	-
Disease activity			
Remission	15 (50%)	53 (49%)	0.792
Low	12 (40%)	38 (35%)	0.546
Moderate-high	3 (10%)	16 (15%)	0.789
CCI	0.5 ± 0.6	0.8 ± 1.4	0.297
AEs after 1 dose *n* (%)	14 (56.7%)	46 (43.4%)	0.938
Serious/severe AEs after 1 dose *n* (%)	0 (0%)	0 (0%)	-
RD relaps after I dose *n* (%)	0 (0%)	4 (3.8%)	0.361
AEs after 2 doses *n* (%)	0 (0%)	20 (31%)	0.253
Serious/severe AEs after 2 doses *n* (%)	0 (0%)	0 (0%)	-
RD relapse after II dose *n* (%)	0 (0%)	0 (0%)	-
**No-Au/cIn-RD**	***n* = 11**	***n* = 37**	
Age (years)	64.1 ± 9.9	67.8 ± 14	0.429
Sex female/male *n*	11/0	32/5	0.319
Disease duration (years)	3.0 ± 2.5	4.0 ± 6.9	0.672
BMI	24.9 ± 2.8	26.1 ± 4.6	0.400
Osteoporosis *n* (%)	4 (36%)	16 (43%)	0.589
Osteoarthrosis *n* (%)	5 (45%)	14 (38%)	0.391
Fibromyalgia *n* (%)	2 (18%)	7 (19%)	0.589
CCI	0.5 ± 0.7	1.2 ± 1.5	0.840
Vaccine second dose	1 (9%)	18 (49%)	0.270
AEs after 1 dose *n* (%)	5 (45%)	12 (33%)	0.660
Serious/severe AEs after 1 dose *n* (%)	0 (0%)	0 (0%)	-
AEs after 2 doses	0 (0%)	4 (20%)	0.140
Serious/severe AEs after 2 doses *n* (%)	0 (0%)	0 (0%)	-

## Data Availability

The data presented in this study are available on request from the corresponding author.
